# On the Use of the Ammonio-tartrate of Iron

**Published:** 1855-09

**Authors:** William Gries

**Affiliations:** Reading, Pennsylvania


*On the use of the Ammonio-tartrate of Iron.* By WILLIAM GRIES, M. D., of Reading, Pennsylvania.

In August, 1851, I received under my care a delicate little girl, 3 years of age, affected with erysipelas over the left hip. She had been, during a year before, very thin in flesh, pale, and much troubled with uneasiness in her bladder, attended with sabulous phosphatic deposit in her urine. When I was requested to attend her for the erysipelas, I found her with high fever and very prostrate. I gave her purgative medicines to correct the secretions of the digestive organs, and soon commenced giving her a solution of ammon. tart. of iron, in 2 gr. doses, every three hours. I had for a long time before this treated erysipelas with sulph. quinia administered freely after mercurial purgatives, and alkaline salts to restore or correct the various secretions. In this case I was prevented from giving the quinia on account of the difficulty there was in giving her any medicine. The comparatively pleasant taste of this preparation was its first recommendation to give it in this case as a tonic, and her previous state of system I thought strongly indicated the use of iron. The duration of the disease was from the 25th of August to the 8th of September. It spread over the whole body, from the left hip down to the foot, and up to the scalp, and thence extended over the whole of the right side. On the parts first invaded it produced vesication, but after twenty-four hours use of the iron I found the extension of the inflammation less and less severe, so that, at last, it was a mere slight erythema. Besides the iron I used no medicine internally, except an occasional small dose of hydrarg. c. c. To prevent the parents from resorting to any external application, I applied, very thinly, tinct. iodine of half the officinal strength. I thought that it might do some good as a counter-stimulant, and not act as a repellent, which I have a terror of, from former sad experience, of which I shall say something in the sequel. As every one knows, erysipelas is a disease of the whole system, and its external manifestation is only the elimination of the poison from the body. My little patient has since enjoyed excellent health, free from every symptom of her former complaint.

On the 31st January, 1852, I received part 24th of Braithwait's Retrospect, in which I found a statement of the remarkable success obtained by G. Hamilton Bell, Esq., in the treatment of erysipelas by the use of the muriated tinct. of iron. When I compared this account with my case, stated above, and considered the favorable report of M. Velpeau, on the local treatment of the disease by sulphate of iron, I concluded that it was the iron introduced into the circulation that was of benefit. Notwithstanding this opinion, when, in February following, I took charge of a severe case of the disease, in a robust man about forty years of age—from the authority above mentioned—I commenced the treatment with the mur. tinct. of iron, but I soon found that my patient could not bear the use of it, in sufficient quantity. It produced a severe burning sensation in his stomach and bowels, attended with vomiting and purging; I therefore substituted the amm. tart. of iron, in 4 gr. doses, every two hours, and soon found so much improvement as to encourage me to continue it, and my patient was soon restored.

Since then I have had the opportunity of using the remedy in this disease very extensively, and always with success, except in two cases of infants within the second week of their birth.

In the latter part of the winter, and in the spring of 1853, I had under my care many cases of typhoid fever, and in every instance I found more or less of inflammation of the pharynx, simulating erysipelas: this I treated, locally, with lunar caustic. I lost two of my patients; and these in consequence of the pharyngitis extending down the œsophagus, producing dysphagia.

Reflecting on the remarkable success I had in the treatment of erysipelas, with the iron, I determined, thereafter, if any case of typhoid fever presented itself with erysipelas, I would give an early trial to this remedy.

It appeared to me that there was sufficient similitude between the two diseases, taking into account the general symptoms, and the peculiar local affection of the glands, in typhoid fever, (I do not pretend to say there is an identity) to warrant the use of it. The proximate principles of its composition are constituents of the body, in its healthy state, and therefore not poisons to it, as some of the prominent remedies recommended, such as superacetate of lead, sulphate of copper or nitrate of silver.

I have searched much for authority to use the iron according to my views, but could find nothing but warning, against the use of it in any disease where there were remains of inflammation in the stomach and bowels, and this is the condition in typhoid fever. When our old treatises on Materia Medica were written, the ordinary preparations of iron in use were the carbonate, the muriated tinct. and the sulphate; the first is objectionable on account of its bulk and its earthy parts, and the two latter are caustic irritants; whence these warnings; and later writers have generally copied these. The ammon. tart. is perfectly soluble, of little taste or irritating properties.

When I reflect that mineral solutions are the chief remedies in inflammation (when the acute stage is passed) of the mucous membrane, wherever we can reach it—in the eye, mouth, vagina, urethra, &c.—I feel a confidence that they may be applied to the same membrane in the stomach and bowels, under the same circumstances, when the articles are of such a nature that we rather desire than dread their absorption.

Since the latter part of last April, a considerable number of cases of typhoid fever have come under my care, generally of a mild character; and one of the first was a robust man about 40 years of age, a carpenter by trade. It came on in such an insidious manner that he thought it was "only a cold," and that he could work it off by taking some patent purgative pills, and continuing at his trade. I was not called till about the tenth day of the disease, when I found him very prostrate. I gave him dilute sulphuric acid, occasionally a dose of blue pill, and ordered ale and porter to be used pretty freely; but in a few days he got an aversion to these drinks, and I substituted brandy and water with sulph. quinia, but neither seemed to agree with him; then I carried out my theorizing, and withheld all other medicines, and commenced with the iron, four grains every four hours; in 48 hours I found such a marked improvement that I felt warranted to persevere. Moreover, my patient expressed himself, decidedly, that this medicine did him more good than any that he had taken. I have, since then, applied it in every case, after using some mercurial purgative of a mild character and dose, and the dilute sulph. acid, until the pungent dry heat of the skin had somewhat abated, although the tongue was furred and dry. Where there was marked epigastric tenderness I first had leeches or cups applied, followed afterwards, during the use of the iron, by poultices sprinkled with spirits of turpentine.

Among all the cases presented to me this season, only two had any thing of external erysipelas, and one of these was in the hands of another practitioner for some time, with severe phlegmonous erysipelatous inflammation of the left wrist and hand, the result, as was supposed, of a puncture of a needle at the wrist, part of which had broken off and remained imbedded. When I saw the case I felt persuaded that it was idiopathic typhoid fever, and that this state of the system determined the unhealthy character of the inflammation. The hand looked nearly gangrenous; it had been lanced at three different places, and I opened a deep seated abcess on the back of the hand, which discharged ichorous pus freely. We agreed to apply to it a poultice of linseed meal and brewer's yeast, and to give the iron internally. The general health and the local affection soon improved, and the patient recovered rapidly.

My own conviction is, that all my typhoid fever patients convalesced and recovered much more pleasantly and rapidly than under any other treatment that I had ever pursued before. Its ordinary effect upon the bowels is to constipate, but it has appeared to me, that in this fever, where there is a tympanitic condition of the bowels, it purges at first; and this, I believe, is in consequence of its tonic effect upon their structure, for the tympanitis disappears with the purging.

In 1837, between the 26th of January and the 13th of April, I had in one family five patients with typhoid fever. First, the father and son were taken; then another son; then a hired girl; and, at last, the mother, who had attended on all the others. The four first all recovered, but the last died on the eleventh day of her sickness. (I write from memory and my account book.) A few days after the invasion of her disease erysipelas appeared on one side of her face, to which I applied a solution of bichloride of mercury, two grains to the ounce of water; in twenty-four hours the inflammation had receded from the face and fixed on the pharynx and posterior nares; I applied an epispastic on the back of the neck, but the inflammation extended through the whole of the cavities of the nose, and, as I had sufficient cause to believe, into the ethmoidal and sphenoidal cells, accompanied with such a profuse flow of irritating mucus from the posterior nares as frequently threatened to strangle her. The inflammation extended to the membranes of the brain, followed by convulsions and death. This determined me *never to use repellents in erysipelas.*

Since I have commenced the use of this preparation of iron, I have cured, with it alone, two severe cases of chorea; one, a girl of 11 years, and the other of 13 years. I have used it also in several cases of disease of the joints, with marked benefit, and in diseases of females, such as chlorosis, neuralgia, and all cases of debility, preferring it to all other tonics, in a general way. In order to make some estimate of the extent of my use of it, I may state, that I have used at least eight pounds in my practice alone; and my practice is not very extensive, for on account of my own bodily infirmities I have to refuse many calls.

In a few cases of intermittent fever I have given it to prevent relapses, and to all appearance with benefit. Though of this I would not speak very positively, because I have, during this period, not had many cases to treat.

My mode of administering it is dissolved in water, either simple or aromatic; ordinarily I use cinnamon water, where not contra-indicated. It makes a perfect solution, having all the physical properties desirable in a medicine. Others may find other preparations of iron equally applicable and effective; I will not dispute with them. I have occasionally, but very seldom, combined with this solution chloric ether, hydrocyanic acid, tincture hyoscyamus, &c., chiefly in severe hysteria.

I have repeatedly mentioned my use of this remedy before our County Medical Society, but I am not aware that more than one of our members has used it, and his experience fully corroborates mine, as far as it goes. I have no doubt that if it had the prestige of some professor's name to commend it, it would have attracted more attention.

I feel persuaded that more than one, reading this, will exclaim, "Riding his hobby to death." I will not be offended with him, for, I fear, I should do the same under similar circumstances. Who has not his hobby? It must be he that cannot ride at all.

